# Increased Engagement, Mixed Outcomes: A Systematic Review of Artificial Intelligence-Based Sleep Interventions for Neurological and Neuropsychiatric Disorders

**DOI:** 10.1192/bjo.2026.11129

**Published:** 2026-06-30

**Authors:** Jake Smith, Yahyee Mohamud, Freya Stephenson, Sarah Allester, Jessica Sidratul

**Affiliations:** King’s College London, London, United Kingdom

## Abstract

**Aims:**

This systematic review aims to synthesize the current literature on Artificial Intelligence (AI)-based interventions for improving sleep outcomes in individuals with neuropsychiatric conditions, excluding those with a primary diagnosis of insomnia.

**Methods:**

This paper is a systematic review conducted in accordance with the PRISMA 2020 guidelines, with two independent reviewers conducting all stages using a double-blind method and conflicts being resolved after each section. A comprehensive electronic search was performed across EMBASE, Medline and PubMed using MeSH terms for Artificial Intelligence, Sleep and Neurological/Neuropsychiatric Conditions. Studies included were English-language, peer-reviewed primary research published between 2022 and November 2025, focusing on AI-based interventions for sleep outcomes in individuals with neuropsychiatric conditions (excluding primary insomnia). Data extraction used the Joanna Briggs Institute (JBI) systematic review framework.

**Results:**

Five studies met the inclusion criteria with wide variations in study type. Sample sizes ranged from 65 to 540 across studies conducted in the UK, USA and Australia. The interventions ranged from AI-based chatbots, mobile apps, wearable devices and sociallyassistive robots. Positive and statistically significant increases in participant engagement were observed, with two papers reporting interactive AI groups attending up to two additional sessions on average (b=1.65, p<0.001) and using the intervention up to 3.8 times longer (p<0.01) compared to the control group. However, overall sleep-related outcomes were mixed; the intention-to-treat analysis in the US randomised control trial (Paper 4) found no statistically significant difference, but post hoc analysis showed significant improvements in sleep quality in the high-device adherence subgroup. Conversely, a cluster randomised control trial (Paper 5) showed no evidence of improved sleep patterns but did note a significant reduction in daytime and night-time motor activity.

**Conclusion:**

Positive and statistically significant increases in participant engagement were observed with the use of artificial intelligence interventions. However, the increased engagement did not result in statistically significant improvements in overall sleep outcomes. The effectiveness of AI interventions is limited by the lack of robust research and universally low sample sizes. Whilst AI interventions have shown to improve engagement, there is currently insufficient evidence to suggest AI interventions improve sleep outcomes in the population group studied.

